# DNA Nanotechnology for Cancer Diagnosis and Therapy

**DOI:** 10.3390/ijms19061671

**Published:** 2018-06-05

**Authors:** Tianshu Chen, Lingjie Ren, Xiaohao Liu, Mengru Zhou, Lingling Li, Jingjing Xu, Xiaoli Zhu

**Affiliations:** 1 Center for Molecular Recognition and Biosensing, School of Life Sciences, Shanghai University, Shanghai 200444, China; s576591524@163.com (T.C.); mickyderen0406@163.com (L.R.); shander5678@126.com (X.L.); m18916571170@163.com (M.Z.); linglingli_007@163.com (L.L.); 2 Institute of Biomedical Engineering, School of Communication and Information Engineering, Shanghai University, Shanghai 200444, China

**Keywords:** DNA nanotechnology, cancer, tumor cells, detection, drug delivery

## Abstract

Cancer is one of the leading causes of mortality worldwide, because of the lack of accurate diagnostic tools for the early stages of cancer. Thus, early diagnosis, which provides important information for a timely therapy of cancer, is of great significance for controlling the development of the disease and the proliferation of cancer cells and for improving the survival rates of patients. To achieve the goals of early diagnosis and timely therapy of cancer, DNA nanotechnology may be effective, since it has emerged as a valid technique for the fabrication of various nanoscale structures and devices. The resultant DNA-based nanoscale structures and devices show extraordinary performance in cancer diagnosis, owing to their predictable secondary structures, small sizes, and high biocompatibility and programmability. In particular, the rapid development of DNA nanotechnologies, such as molecular assembly technologies, endows DNA-based nanomaterials with more functionalization and intellectualization. Here, we summarize recent progress made in the development of DNA nanotechnology for the fabrication of functional and intelligent nanomaterials and highlight the prospects of this technology in cancer diagnosis and therapy.

## 1. Introduction

Cancer, a major and increasing health problem worldwide, has already become the second leading cause of death in recent years. As a major, yet unmet threaten to healthcare globally, cancer causes about 9 million deaths each year in the whole world [1,2]. In order to increase the survival rate of cancer patients, early diagnosis and timely therapy become extremely essential to improve the prognosis of cancer patients, especially breast cancer patients. However, current diagnostic technologies, including imaging, molecular detection, immunohistochemistry (IHC), and so on, have inherent limitation, such as a potentially lower accuracy [3]. In addition, for cancer therapy, researchers have been constantly improving anti-cancer drug delivery systems to target tumor cells or tissues more accurately and produce less side effects than chemotherapy [4]. However, current progress can not satisfy the increasing demand for more effective and highly biocompatible drug delivery systems [5].

**Figure 1:**
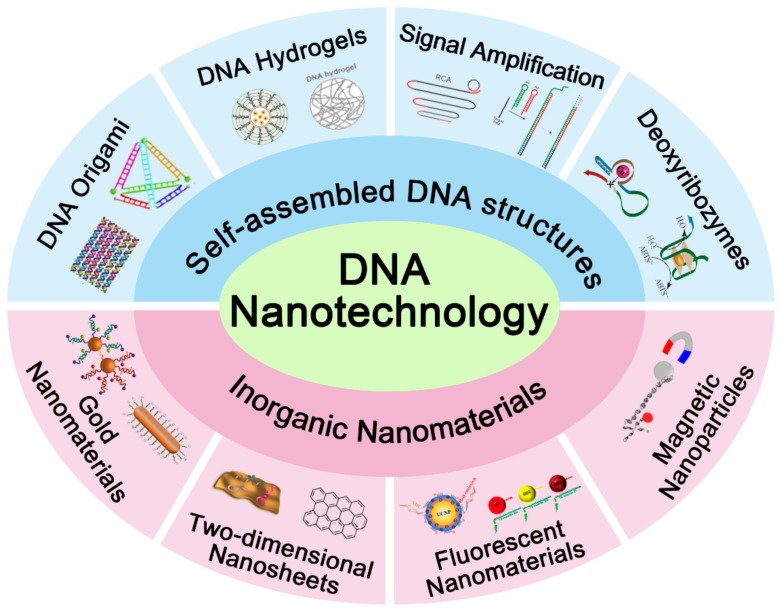
Schematic illustration of different applications associated with DNA nanotechnology.

In order to overcome the challenges aforementioned, deoxyribonucleic acid (DNA) has drawn a lot of attention, owing to its predictable secondary structure, small size, high biocompatibility, and programmability [6]. Moreover, DNA nanotechnology, a technique applying the biomolecular self-assembly property of DNA, has a wide range of applications in various disciplines, especially in synthetic biology, chemical analysis, and drug delivery [7]. Upon the formation of specific base pairs, DNA strands hybridize with each other and can then be easily engineered into a functional nanostructure with highly spatial programmability [8,9,10,11], such as designed DNA nanodevices compatible with the immune system and DNA-based smart drug-delivery vehicles [12,13]. As a promising diagnostic and therapeutic nanoplatform, DNA strands combined with other nanoscale materials, such as nanowires, nanotubes, nanosheets, polymers, gold nanoparticles (AuNPs), quantum dots, and iron oxides, show a great potential in early diagnosis of cancer and timely therapy [14,15]. This review summarizes recent progress in the development of DNA nanotechnology as shown in Figure 1 and deals with the application of DNA nanotechnology in synthesizing functional and intelligent nanomaterials for cancer diagnosis and therapy.

## 2. DNA Nanotechnologies for the Establishment of Theranostic Nanoplatforms

As a natural molecule, DNA can be used to build functional nanostructures through adenine (A)–thymine (T) and guanine (G)–Cytosine (C) Watson–Crick base pairing (bp) [16,17,18,19]. Followed by specific base pairing, DNA self-assembly can acquire properties, such as molecular recognition and assembled structure construction, which may be utilized in a variety of applications, including targeted cancer diagnosis and therapy [20,21]. In this section, DNA nanotechnology based on various self-assembled structures of DNA is introduced and the performance of DNA-based nanostructures for cancer diagnosis and therapy is discussed.

### 2.1. DNA Origami-Based Theranostic Nanoplatform

DNA origami, a self-assembled structure, is capable of localizing DNA hybridization reactions on two-dimensional (2D) lattices or three-dimensional (3D) self-assembled nanostructures. DNA origami nanostructure is regarded as a nanoplatform that can provide opportunities to develop a large number of applications, including biosensing for cancer diagnosis and drug delivery for cancer therapy [22,23,24,25,26].

DNA tetrahedron, a 3D self-assembled DNA origami nanostructure, is widely used as a sensitive biosensing probe which can be rapidly internalized by a caveolin-dependent pathway. Furthermore, DNA tetrahedron can retain the structural integrity within cells over a long period [27,28]. He et al. developed a new strategy using self-assembled DNA tetrahedron for highly reliable detection of tumor-related mRNA. Their detection was based on the fluorescence resonance energy transfer (FRET) “off” to “on” signal readout mode. Specifically, in tumor cells, the DNA tetrahedron that carried dual separated fluorophores displayed low FRET efficiency in the absence of a target mRNA. On the contrary, the presence of a target mRNA brought about proximity of fluorophores, which led to high FRET efficiency [29].

Moreover, DNA origami-based molecular recognition elements can be integrated with anti-cancer drugs to offer specific location information of cancer cells and exert therapeutic effects on cancer at the same time. Doxorubicin hydrochloride (Dox) is well known as an effective anti-cancer drug owing to its strong affinity toward DNA double helix and ability of intercalating into C−G base pairs [30,31], so it is widely used for anti-cancer therapy. Zhang et al. reported a self-assembled DNA origami nanostructure with intercalated Dox, which exhibited remarkable efficacy for cancer therapy. According to imaging results in vitro and in vivo, the DNA origami, especially the triangle-shaped, was observed to transport Dox to tumor cells and tissues and exhibited long-lasting accumulation in the tumor. This was the first time that DNA origami was proved to passively accumulate in the tumor region in an efficient manner [32].

**Figure 2:**
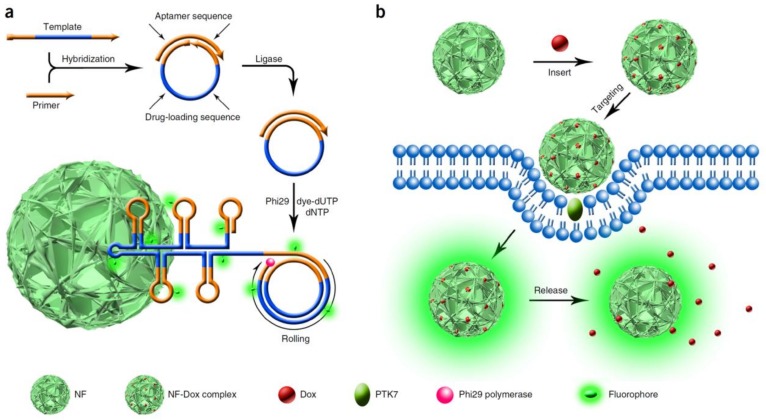
Schematic illustration of noncanonical self-assembly and biomedical applications of multifunctional DNA nanoflowers (NFs). (**a**) Rolling circle amplification (RCA) generates a large amount of elongated DNA, which provides the building blocks to self-assemble the DNA NFs; (**b**) A drug (Doxorubicin, Dox) is loaded into NFs, which are able to target protein tyrosine kinase 7 (PTK7)-overexpressing cells through the incorporated sgc8c aptamer. Dox is released from NFs after intracellular uptake and causes selective apoptosis. dNTP: deoxy-ribonucleoside triphosphate. Reproduced with permission from Ref. [33] Copyright 2015 Springer Nature.

More interestingly, Lv et al. developed a Dox-loaded DNA nanostructure termed nanoflowers (NFs), consisting of self-assembled DNA building blocks generated by rolling circle amplification (RCA) in the presence of a template. As shown in Figure 2, DNA template and primer hybridized with each other and were then ligated and amplified with the help of a ligase and a polymerase. The DNA NFs were multifunctional nanomaterials composed of multiple components, including dye-modified deoxyuridine triphosphate (dUTP) for intracellular imaging, aptamers sgc8c, which could specifically recognize protein tyrosine kinase 7 (PTK7) for cell targeting, and Dox inserted into the C−G base pairs for drug delivery. In fact, the DNA NFs showed exceptional promise and potential for cancer therapy [33].

**Figure 3:**
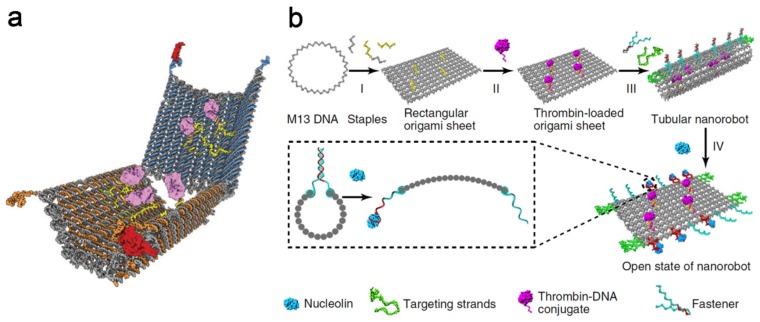
Design of a functional DNA nanorobot. (**a**) Schematic illustration of a DNA nanorobot loaded with molecular cargoes (yellow and pink) and formed by a large viral genome-derived DNA strand (grey) folding with several staple strands (blue and orange) that can be stabilized in a dissociated state by target (red). Reproduced with permission from Ref. [34] Copyright 2012 American Association for the Advancement of Science; (**b**) Schematic illustration of the construction of a thrombin-loaded nanorobot by DNA origami and its reconfiguration into a rectangular DNA sheet in response to nucleolin binding. I, Single-stranded M13 phage genomic DNA is linked by predesigned staple strands, leading to the formation of a rectangular DNA sheet; II, Thrombin is loaded onto the surface of the DNA sheet structure by hybridization of poly-T oligonucleotides conjugated to thrombin molecules with poly-A sequences that extend from the surface of the DNA sheet; III, Addition of the fasteners and targeting strands results in the formation of thrombin-loaded, tubular DNA nanorobots with additional targeting aptamers at both ends; IV, The tube nanocarrier opens in the presence of nucleolin to expose the encapsulated thrombin. Reproduced with permission from Ref. [35] Copyright 2018 Springer Nature.

DNA nanorobot (Figure 3a) is an autonomous cargo delivery vehicle for transporting molecular cargoes to cells, reported by Douglas et al. in 2012 [34]. Afterwards, Li et al. reported a cancer therapy strategy based on a designed autonomous DNA nanorobot for thrombin delivery, in 2018. The growth of tumors depends on a sufficient supply of nutrients and oxygen provided by the tumor blood vessels. This thrombin carried nanorobot could lead to thrombosis in tumor vessels, which then cut off the supplies and eventually caused the death of tumor cells. As shown in Figure 3b, based on the single-stranded M13 phage genomic DNA, the DNA nanorobotic system formed as a rectangular DNA origami sheet containing poly-A oligonucleotides, which could conjugate with poly-T oligonucleotide-modified thrombin. Then, the fastener strands containing DNA aptamers (AS1411) could hybridize with the DNA sheet, thus forming a tube-shaped DNA nanorobot. This system could target nucleolin, a protein which is specifically expressed in tumor-associated endothelial cells, by opening the DNA nanorobot through a mechanical movement in the presence of AS1411. Upon thrombin exposure, thrombosis occurred at the tumor site, leading to the final disappearance of cancer [35].

### 2.2. DNA Hydrogel-Based Theranostic Nanoplatform

Hydrogels are crosslinked hydrophilic polymers that have been widely used as scaffolds for drug delivery and 3D cell culture because of their good biocompatibility, plasticity, and capability of providing 3D scaffolds [36,37]. DNA-based hydrogels have drawn great attention because of the unique features brought by nucleic acids, such as stability, flexibility, precise programmability, switchable properties, and facile synthesis and modification [38]. At present, functional DNA hydrogels re intensively applied in tumor cells analysis and drug delivery.

**Figure 4:**
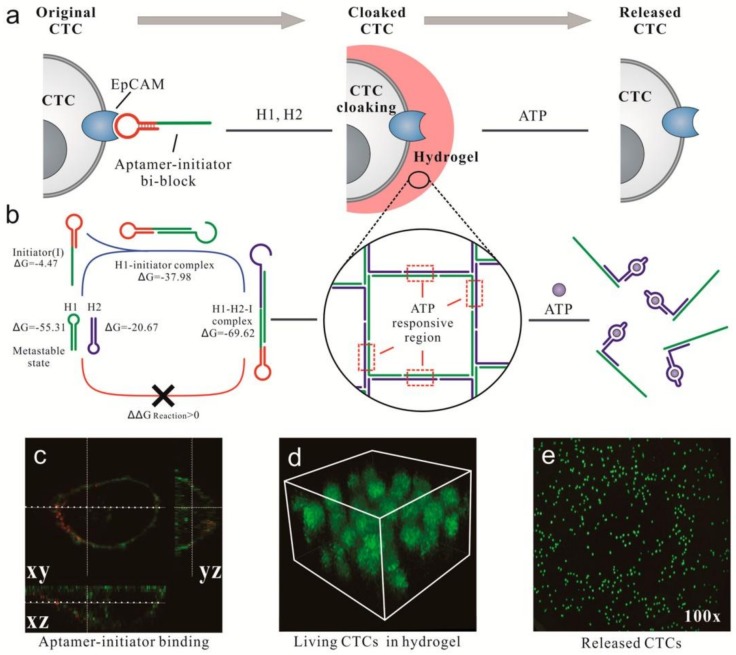
DNA gelation-based cloaking and decloaking of circulating tumor cells (CTCs). (**a**) The aptamer-initiator biblocks (red line) are able to specifically bind the epithelial cell adhesion molecule (EpCAM) on the cell surface, which can then trigger the aptamer-trigger clamped hybridization chain reaction (atcHCR) to assemble the DNA hydrogel. Adenosine triphosphate (ATP) is used to destroy the DNA hydrogel containing ATP-responsive regions (purple line); (**b**) DNA hairpins H1 and H2 were trapped in a metastable state. Without a DNA initiator, H1 and H2 cannot hybridize. With a DNA initiator, H1 is opened and triggers the subsequent hybridization chain reaction; (**c**) Confocal images of aptamer-initiator biblocks (red) colocalized with 3,3′-dioctadecyloxacarbocyanine perchlorate (DiO) stained lipids on the cell membrane (green); (**d**) 3D stack of MCF-7 cells cloaked in a DNA hydrogel with FDA staining in green, showing multilayered cells in the hydrogel. Stack height: 40 μm; (**e**) By adding ATP, MCF-7 cells were released and dispersed in solution. Reproduced with permission from Ref. [39] Copyright 2017 American Chemical Society.

Song et al. designed a system of in situ DNA gelation triggered by aptamers for capturing or releasing a low number of circulating tumor cells (CTCs) in whole blood. In Figure 4, CTCs first combined with aptamer-initiator bi-block strands, which could specifically recognize epithelial cell adhesion molecule (EpCAM), a biomarker protein that was highly expressed on the surface of CTCs. Afterwards, an aptamer-trigger clamped hybridization chain reaction (atcHCR) was triggered to assemble the DNA hydrogel and capture the CTCs. The DNA hydrogel, containing an ATP-responsive region, could be destroyed via a conformational change of the aptamer in the presence of ATP, which led to the release of CTCs. This method is expected to be effective in cancer diagnostics and therapeutics [39].

For cancer therapy, the application of DNA hydrogels is limited because of their large size and lack of efficient release mechanisms. To overcome these challenges, Li et al. developed a size-controllable and stimuli-responsive DNA hydrogel for targeted gene therapy. This DNA hydrogel was generated by assembly of Y-shaped DNA molecules, which were the building units, linking units, blocking units, and targeting units. The disulfide linkage-incorporating hydrogel could be cleaved by glutathione (GSH) in the cytosol of tumor cells, which resulted in the selective release of therapeutic oligonucleotides into the tumor cells [40].

### 2.3. DNA Signal Amplification-Based Theranostic Nanoplatform

Currently, DNA-based signal amplification strategies play an important role in medical diagnostics. The strategies can be divided into two categories: thermocycling amplification strategies, like polymerase chain reaction (PCR), and isothermal amplification strategies, like rolling circle amplification (RCA), hybridization chain reaction (HCR), strand-displacement amplification (SDA), etc. The latter is more suitable than the others for the detection of trace biomolecules on the cell surface or even inside living cells, delivering drugs, and in vivo imaging [41,42,43,44].

**Figure 5:**
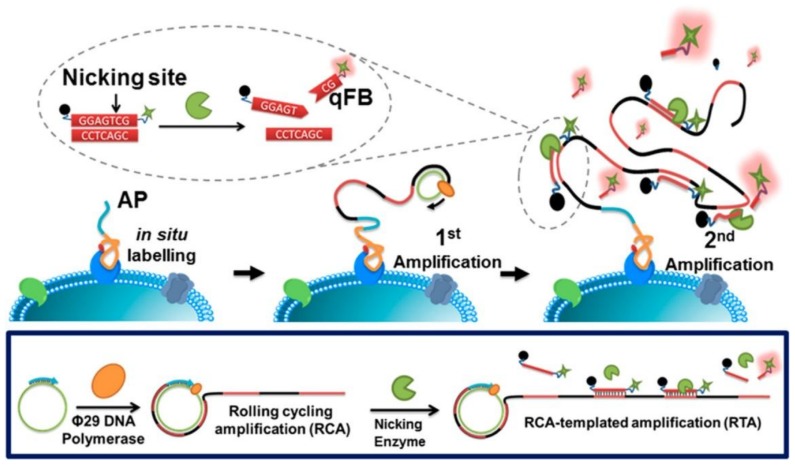
Principle and procedures of in situ rolling cycling replication-templated amplification strategy (isRTA) for plasma membrane proteins (PMPs) quantitation. Reproduced with permission from Ref. [45] Copyright 2017 American Chemical Society.

Gao et al. proposed a novel method for quantitatively analyzing plasma membrane proteins (PMPs) by an in situ rolling cycling replication-templated amplification strategy (isRTA). As shown in Figure 5, two rounds of amplification occur through the cascade of isothermal reactions which were triggered by RCA and a nicking enzyme, sequentially. Then, the PMPs are quantified precisely by florescence measurements. In this work, isRTA was applied to quantify tumor-associated PMP biomarkers (such as MUC1, EpCAM, and HER2), which might reflect different breast cancer phenotypes [45]. In addition, an effective method, termed netlike rolling circle amplification (NRCA), including a nicking enzyme into a hyperbranched RCA system, was developed by Zhu et al. [46]. This method was successfully applied to quantify a miRNA (miR-21) in cancer cells [47]. Recently, the same group reported an immunoassay based on RCA, named immuno-NRCA, for the ultrasensitive detection of tumor biomarkers. Different from the conventional enzyme linked immunosorbent assay (ELISA), this immuno-NRCA first detected the targets on a 2D interface, and then the reaction was transferred to a 3D solution for signal amplification [48].

### 2.4. Other DNA Nanotechnologies for the Establishment of Theranostic Nanoplatforms

The fundamental sequence of DNA dictates the structural and reactive features of the biopolymer. For instance, G-rich sequences self-assemble into G-quadruplexes in the presence of K^+^ or NH^4+^, C-rich strands transform into i-motifs under pH-stimulation, and bases–ions form, such as T–Hg^2+^–T or C–Ag^+^–C bridges [49,50]. Furthermore, the catalytic deoxyribozymes (DNAzymes) have the ability to yield catalytic nucleic acid nanostructures by acting as chelators for the association of ions [51,52,53]. DNA functional nanostructures can assemble upon stimulation, a property that can be used for sensing and drug release.

**Figure 6:**
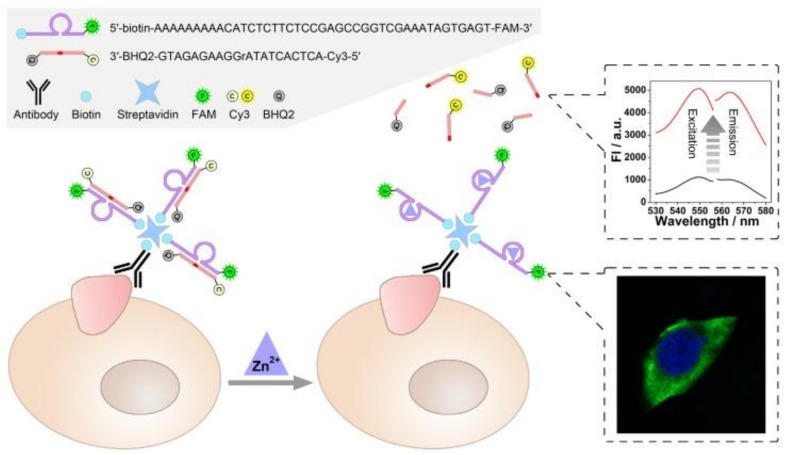
Scheme of the DNAzyme-based nondestructive analysis of tumor-associated membrane proteins (TMPs) via cell imaging and signal amplification. Reproduced with permission from Ref. [54] Copyright 2018 Ivyspring International Publisher.

Chen et al. developed a facile method for in situ imaging and quantification of tumor-associated membrane proteins (TMPs) in the same system, based on fluorophore-labeled DNAzyme. As shown in Figure 6, a zinc ion-dependent biotin-modified DNAzyme that carried a fluorophore (FAM) was conjugated with biotin-modified TMPs antibodies on the cell surface through streptavidin–biotin conjugation, hybridized, and then cleaved, releasing the substrate strand of the DNAzyme that carried a fluorophore (Cy3) and a quencher (BHQ2) at its 5′- and 3′-terminal respectively. In this way, the expression level of TMPs in situ as well as their locational information could be visualized. In addition, a quantitative analysis of TMPs could be achieved by the catalytic activity of the DNAzyme, which then provided fluorescent signals [54]. Based on the functional DNA nanostructure, a pH-responsive dynamic i-motif DNA nanocluster that provided tumor targeting and drug loading capabilities was designed by Kim et al. They integrated gold nanoparticles modified with antisense and i-motifs of DNA sequences, which were then applied as drug delivery vehicles for tumor-targeting therapy [55].

## 3. DNA-Integrated Gold Nanomaterials for the Establishment of Theranostic Nanoplatforms

In recent years, gold nanomaterials, such as gold nanoparticles (AuNPs) and gold nanorods (AuNRs), have shown great potential for targeting and drug delivery. Their wide applications can be attributed to their advantages, such as ease of synthesis, controllable shape, and tunable surface functionalities [56]. Their well-known optical properties endow them with ideal performance for medical imaging and even for photothermal therapy in the field of cancer diagnosis and therapy [14]. In this section, we summarize the applications of DNA-integrated gold nanomaterials for cancer cells imaging and drug delivery.

### 3.1. DNA-Integrated AuNPs

AuNPs are among the most extensively studied nanomaterials, because of their high stability, especially their extraordinary optical and electrical properties [57]. AuNPs can be easily coupled with DNA by covalent binding or electrostatic interaction to obtain receptors specifically targeting biomarkers on cancer cells [58].

**Figure 7:**
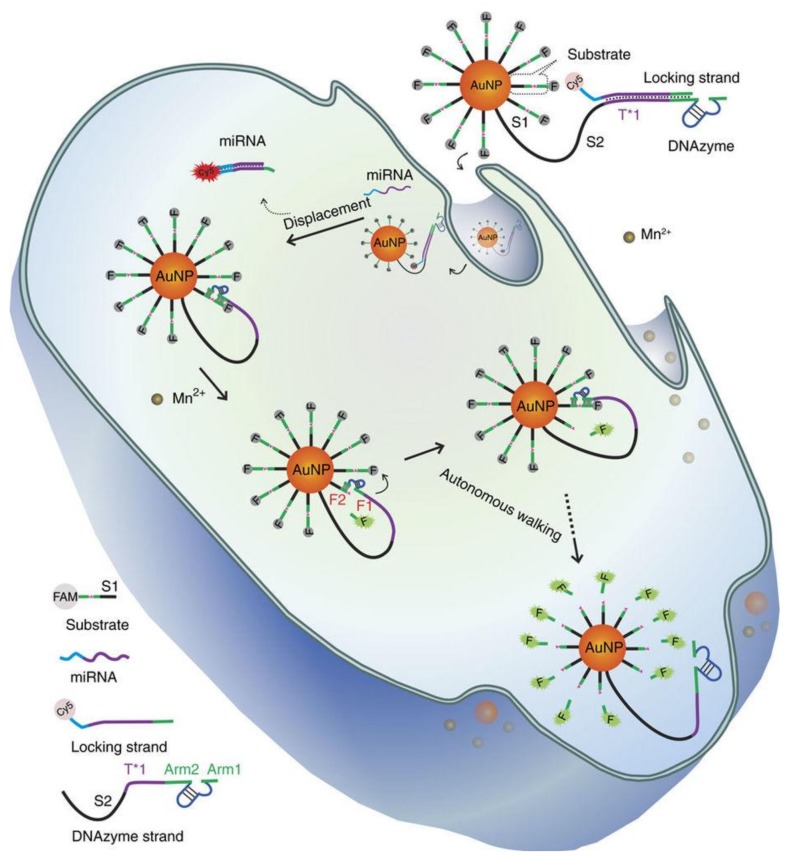
Schematic representation of the intracellular operation of a DNAzyme motor initiated by a specific miRNA. Reproduced with permission from Ref. [60] Copyright 2017 Springer Nature.

Recently, a signal amplification strategy has been established for DNA walking nanomachine-combined AuNPs [59]. For instance, Peng et al. constructed a DNAzyme motor on a 20 nm AuNPs for real-time detection of a specific miRNA in individual cancer cell. As shown in Figure 7, AuNPs were integrated with dozens of DNAzyme strands (complementary towards locking strands) and hundreds of substrate strands. For imaging purposes, the locking strands and substrate strands were labelled with cyanine5 (Cy5) and carboxyfluorescein (FAM), respectively, which wold be quenched by AuNPs in the absence of a target miRNA. In the presence of a target miRNA in living cells, the DNAzyme could cleave the substrate strands one by one, which resulted in the autonomous walking of the motor on the AuNPs. In the end, the fluorophore-labelled DNA strands on AuNPs were released, allowing real-time imaging of the intracellular miRNA [60]. Differently, Wu et al. developed a new DNA nanoassembly by generating a thin layer of cationic peptides onto AuNPs. Specifically, the nanoparticles were loaded with fluorophore-labeled hairpin DNA probes, which could ultra-sensitively show mRNA in living cells via HCR. This example provided an invaluable platform for highly sensitive detection of low-abundance intracellular biomarkers in clinical diagnostics [61].

In addition, it has been reported that DNA nanotechnology established on AuNPs is extremely suitable for drug delivery. Ma et al. designed a molecular beacon-based drug delivery system for telomerase-triggered drug release for cancer therapy. The fluorescein isothiocyanate (FITC)-labeled DNA hairpins that could hybridize with telomerase primers and the anticancer drug Dox were both loaded on AuNPs. This system could specifically distinguish normal cells and tumor cells, treat cancer cells on the basis of telomerase activity, and detect telomerase activity in living cells [62].

### 3.2. DNA-Integrated AuNRs

**Figure 8:**
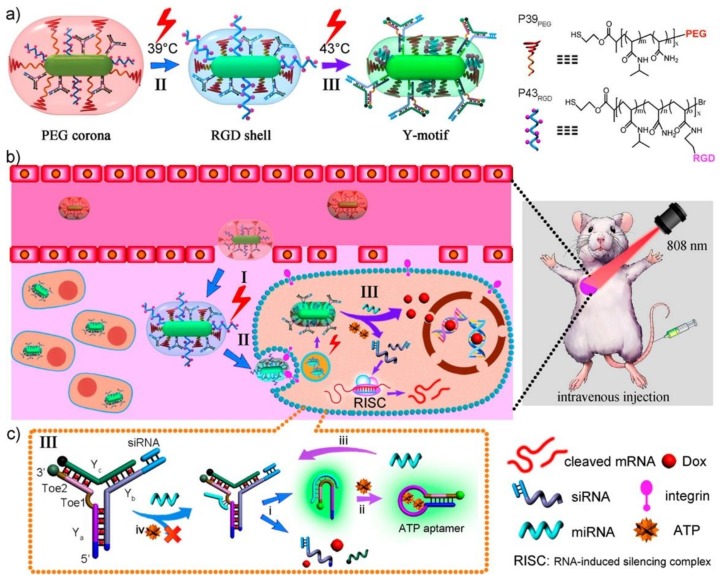
Schematic design of smart nanocarriers triggered by miRNA and fueled by ATP. (**a**) Thermal-responsive regulation of the surface composition; (**b**) Near-infrared (NIR)-guided delivery and controlled release in vivo. I, passive accumulation at tumor sites via enhanced permeability and a retention (EPR) effect; II, specific uptake via receptor-mediated endocytosis; III, controlled release by endogenous miRNA/ATP; (**c**) Amplified dissociation of Y-motifs based on toehold-mediated strand displacement (TMSD) cascade reactions with miRNA as the trigger and ATP as the fuel: i, Toe1-TMSD triggered by miRNA; ii, ATP (or H^+^) recognition by aptamer; iii, Toe2-TMSD for miRNA regeneration. Reproduced with permission from Ref. [66] Copyright 2016 American Chemical Society.

AuNRs have excellent photothermal conversion properties upon light irradiation, especially with near-infrared (NIR) light (650–900 nm), and thus have been widely used in photothermal therapy (PTT) and photodynamic therapy (PDT) [63,64]. Using the photothermal conversion ability of AuNRs, Dai et al. integrated some programmable DNA hairpin probes onto AuNRs, which could then couple with target strands. During the reaction, displacement and amplification were triggered by NIR light for miRNA imaging and quantification in tumor cells and multicellular tumor spheroids. This is an innovative way for sensitive and real-time detection of miRNA in single living cells [65]. Moreover, Zhang et al. designed a smart nanocarriers by assembling stimuli-responsive Y-motifs DNA and two thermosensitive polymers (P39_PEG_ and P43_RGD_) onto the surface of AuNRs for targeted delivery and controlled release of siRNA and Dox in vivo. As shown in Figure 8, AuNRs heated the surrounding environment over 39 °C under mild NIR irradiation, which led to the shrinkage of P39_PEG_ and the exposure of P43_RGD_, then facilitated the specific receptor-mediated endocytosis by tumor cells. Then, the dissociation of the Y-motifs was triggered by the endogenous miRNAs in the presence of ATP, followed by the release of Dox and siRNA, the synergetic induction of cell apoptosis, and the inhibition of tumor growth with limited side effects in normal tissues. This work provides a promising platform for the design of smart nanocarriers, which are DNA nanomachine-functionalized AuNRs and polymers, for targeted cancer therapy [66].

## 4. Other Materials Integrated with DNA

### 4.1. 2D Nanosheets Integrated with DNA

**Figure 9:**
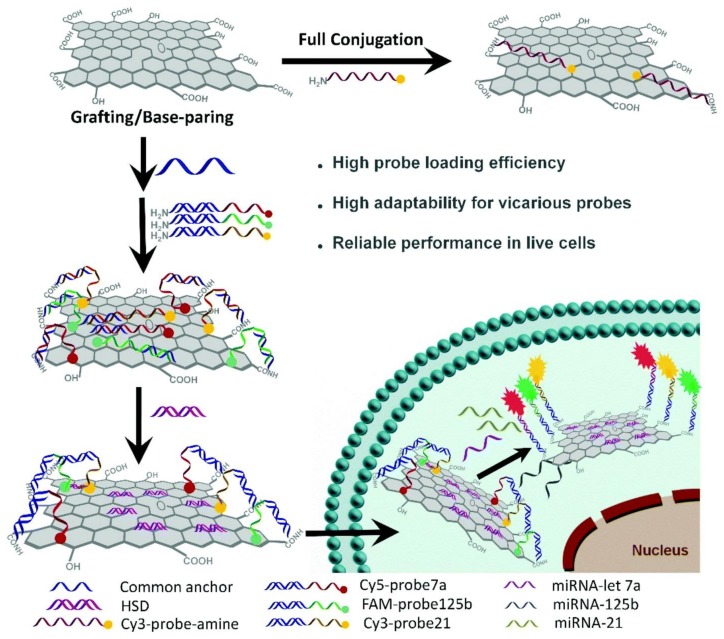
Schematic illustration of two different strategies for constructing nanosystems for miRNA detection, i.e., the full conjugation strategy and the graft/base-pairing strategy mediated by a common anchor sequence. Reproduced with permission from Ref. [70] Copyright 2018 Royal Society of Chemistry.

As a 2D nanosheet structure, graphene or graphene oxide (GO) is a single layer of graphite, which is composed of well-arranged carbon atoms. In order to be applied in clinical diagnosis and cancer therapy, the 2D nanosheets are usually modified with DNA, because of graphene excellent DNA adsorption ability, easiness of functionalization, surface enhanced Raman scattering (SERS) property, and fluorescence quenching ability [67,68,69]. Yu et al. developed a GO–DNA nanosystem to facilitate the detection of intracellular multiplex microRNAs. As shown in Figure 9, the common anchor sequence of DNA was conjugated onto GO surface via 1-(3-dimethylaminopropyl)-3-ethylcarbodiimide hydrochloride (EDC)/*N*-hydroxysuccinimide;1-hydroxypyrrolidine-2,5-dione (NHS) coupling, this obtaining a GO–DNA nanosystem. This system could specifically differentiate miRNAs in living cells, with high stability in a complex biological environment, high resistance to nuclease, and no cross-reaction [70]. Beside GO, MnO_2_ nanosheets have attracted extensive attention in bioanalysis, cell imaging, and drug delivery, because of their appealing physicochemical properties. Fan et al. developed some MnO_2_ nanosheets integrated with DNAzyme for gene silencing therapy. In cancer cells, intracellular glutathione (GSH) can oxidize MnO_2_ to Mn^2+^ ions, which serves as cofactors for DNAzyme to cleave mRNA and perform gene silencing [71].

### 4.2. Fluorescent Nanoparticles Integrated with DNA

Recently, semiconductor quantum dots (QDs) have been widely used as a new class of fluorescent nanomaterials. The high quantum yield and fluorescence signal properties of QDs made them suitable for cancer cells imaging [72,73]. Li et al. developed a strategy using DNA copolymerized QDs for ultrasensitive imaging of specific cancer cell types. Specifically, some aptamers were coupled with QDs through hybridization chain reaction (HCR) to constitute linear QD–aptamer polymers (QAPs). Using this system, the authors obtained a strong fluorescent signal from cancer cells with high resolution, by using QDs at very low concentrations (as low as 5 nM). Moreover, accurate and sensitive detection of single cells, such as circulating tumor cells (CTC), has also been achieved [74].

**Figure 10:**
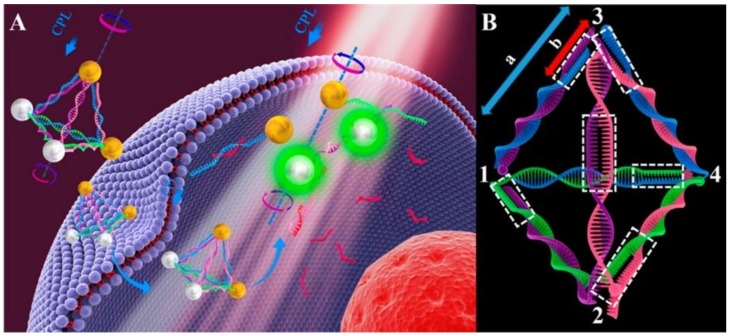
(**A**) Principle of Au–Upconversion nanoparticles (UCNP) pyramids for miRNA detection; (**B**) The nucleic acid skeleton of the pyramid used for miRNA detection. 1, 2 are linked with UCNPs; 3, 4 are linked with Au NPs; a, recognition sequence of miRNA; b, non-complemented part. CPL: circularly polarized light. Reproduced with permission from Ref. [76] Copyright 2016 American Chemical Society.

Upconversion nanoparticles (UCNPs), another kind of fluorescent nanomaterials better than conventional fluorescent dyes, exhibit excellent physio-chemical properties, including excitation with near-infrared light, anti-Stokes emission, superior photostability and chemical stability, low toxicity, high signal-to-noise ratios, etc. Modification of UCNPs by using DNA nanotechnology may facilitate biological detections [75]. Li et al. developed a DNA-driven self-assembled pyramid which was composed of AuNPs and UCNPs. This pyramid could output dual-mode signals to achieve ultrasensitive and highly selective detection of miRNA in live cells. As shown in Figure 10, four recognition sequences were inside the DNA frame and could promote the assembly of AuNPs and UCNPs (termed as Au–UCNP pyramids). The pyramid could display strong plasmonic circular dichroism (CD) at 521 nm and significant luminescence in 500−600 nm, owing to the unique optical properties of UCNPs. Between the AuNPs and the UCNPs, the luminescence in the pyramids was quenched by the luminescence resonance energy transfer (LRET). This work provided a new method for ultrasensitive and highly selective detection and quantification of miRNA in living cells [76].

### 4.3. Magnetic Nanoparticles Integrated with DNA

**Figure 11:**
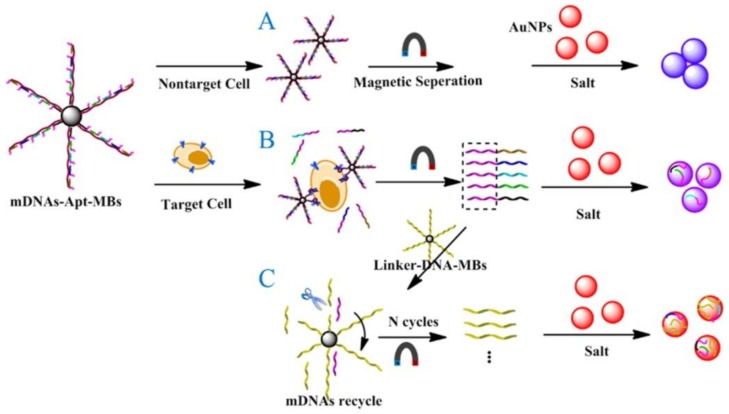
Schematic representation of the visual detection of HL-60 cells based on aptamer DNA conformational switch and non-crosslinking AuNPs aggregation: (**A**) Blank; (**B**) Conformational switch of mDNAs–Apt–MBs; (**C**) Cyclic enzymatic amplification. Reproduced with permission from Ref. [80] Copyright 2016 American Chemical Society.

Magnetic nanoparticles (MNPs) play an important role in various biomedical applications, such as biosensing, drug delivery, medical imaging, cancer therapy, and magnetic separation, because of their strong magnetic properties [77,78]. The magnetic properties of MNPs depend significantly on particle size distribution and shape [79]. To perform real-time detection and treatment with high accuracy, MNPs are often coupled with DNA as targeting agents. Yu et al. developed an easy and intuitive dispersion-dominated colorimetric strategy for cancer cell detection based on a kind of aptamer–magnetic bead bioconjugate. As shown in Figure 11, the aptamer–magnetic bead bioconjugate (mDNAs–Apt–MBs) was fabricated by grafting five kinds of messenger DNAs (mDNAs) aligned with the cancer cell aptamers onto MNPs. In the presence of target cancer cells, mDNAs–Apt–MBs could bind to these cancer cells via the aptamers and then release mDNAs. After magnetic separation, the released mDNAs underwent cyclic enzymatic amplification and produced more single strand DNA (ssDNA) fragments. Through the ssDNA fragments and mDNAs adsorbed on the surface of AuNPs, a novel, simple, and ultrasensitive colorimetric method for cancer cell detection was achieved [80].

## 5. Conclusions and Perspectives

The applications of DNA nanotechnology and of DNA nanotechnology-improved nanomaterials were reviewed in this paper. Through Watson–Crick base pairing, DNA can self-assemble to become a functional nanostructure, which has been widely used for cancer cells imaging and targeted drug delivery. Moreover, by employing a variety of nanomaterials, such as gold nanomaterials, nanosheets, fluorescent nanoparticles, and magnetic nanoparticles, DNA can be more efficiently utilized in cancer diagnosis and therapy, with reduced risk to be degraded by intracellular nuclease. In the near future, it is certain that more and more functional and intelligent nanomaterials based on DNA will be developed for cancer diagnosis and therapy. Despite the inherent disadvantages of DNA-based nanomaterials, such as induction of undesired immune responses, poor efficacy of transport, and poor stability in the tumor environment, great advances have already been made to overcome these challenges so to achieve early tumor diagnosis and precise treatment in a near future. In the meantime, it is clear that more efforts should also be devoted for the improvement of the safety and stability of DNA-based nanomaterials.
